# Steroid‐responsive aseptic meningitis with raised intracranial pressure syndrome associated with myelin oligodendrocyte glycoprotein autoantibodies

**DOI:** 10.1111/jpc.16189

**Published:** 2022-08-24

**Authors:** Wui‐Kwan Wong, Christopher Troedson, Ken Peacock, Fabienne Brilot‐Turville, Manoj P Menezes, Russell C Dale, Richard Webster

**Affiliations:** ^1^ TY Nelson Department of Neurology and Neurosurgery The Children's Hospital at Westmead Sydney New South Wales Australia; ^2^ CHW Clinical School, Sydney Medical School, Faculty of Medicine and Health University of Sydney Sydney New South Wales Australia; ^3^ Department of General Paediatrics The Children's Hospital at Westmead Sydney New South Wales Australia; ^4^ Brain Autoimmunity Group, Kids Neurosciences Centre Kids Research at the Children's Hospital at Westmead Sydney New South Wales Australia

**Key points**

1. Aseptic meningitis may be associated with anti‐myelin oligodendrocyte glycoprotein (MOG) antibody‐associated inflammatory encephalitis.
2. Anti‐MOG‐associated aseptic meningitis is extremely steroid‐responsive and treatment with high‐dose steroids can result in complete recovery.
3. Testing for anti‐MOG antibodies should be considered in children with unexplained aseptic meningitis.

Aseptic meningitis is a syndrome of meningitis with a negative gram stain and bacterial culture.^1^ Aetiologies include viruses, fungi, parasites, post‐infectious causes, drugs, systemic disease and malignancy.^1^ Identifying the cause of aseptic meningitis is often challenging.

Anti‐myelin oligodendrocyte glycoprotein (MOG) antibodies are associated with several steroid‐responsive paediatric neuroinflammatory diseases, with acute disseminated encephalomyelitis (ADEM) being the commonest in children.^2^ Recently, aseptic meningitis has been described as a presentation of anti‐MOG antibody‐associated disease (MOGAD) in childhood.^3^, ^4^, ^5^ Given the steroid‐responsive nature of MOGAD, it is an important diagnosis to consider paediatric aseptic meningitis.

We report three children who presented with aseptic meningitis and raised intracranial pressure (ICP) who progressed to ADEM and other encephalomyelitis phenotypes 2–5 weeks later; these patients were found to be MOG antibody positive.

## Case Reports

### Patient A

**Fig. 1:**
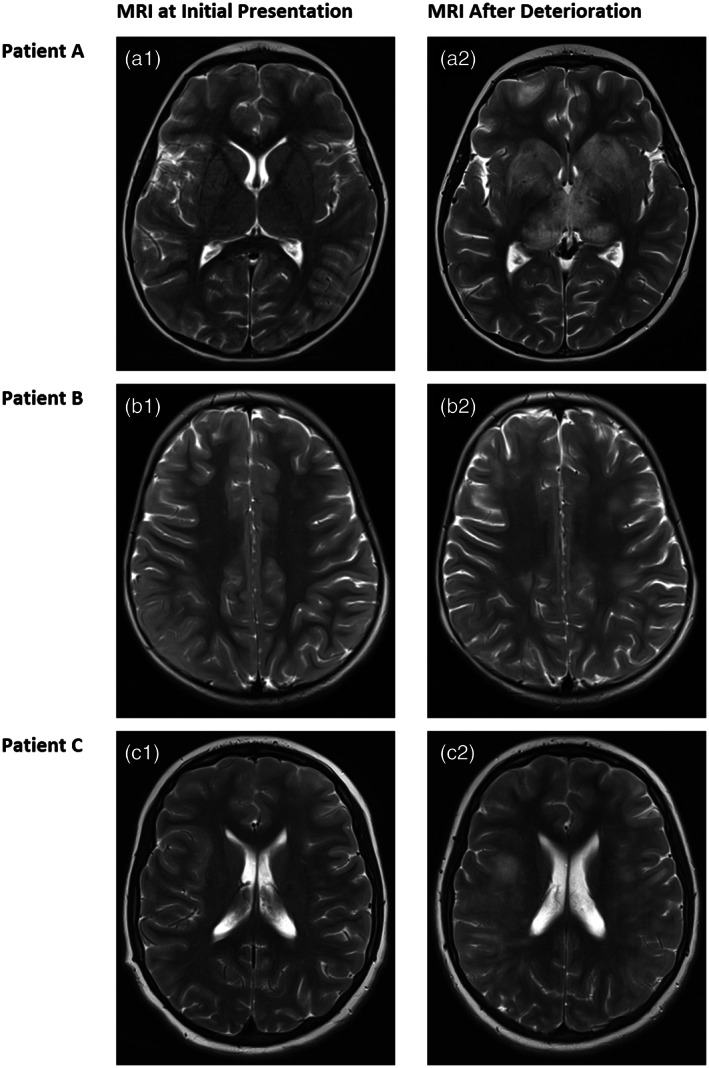
Comparative T2 MRI images in initial presentation (a1, b1 and c1) and after deterioration (a2, b2 and c2) of patient A (a1 and a2), patient B (b1 and b2) and patient C (c1 and c2). Initial MRI images are normal or showing subtle changes only with repeat MRI showing clear inflammatory changes in the deep grey and white matter regions. Axial brain MRI images after deterioration show multifocal, poorly defined T2 high signal in deep grey matter of patient A, multifocal, poorly defined T2 high signal throughout the white matter and cortex in patient B and white matter T2 high signal in patient C. MRI, magnetic resonance imaging.

Patient A was a 5‐year‐old female who presented with fever and headache but no encephalopathy or focal neurological signs. Cerebrospinal fluid (CSF) showed 6 × 10^6^/L polymorphs (normal < 1 × 10^6^/L), 10 × 10^6^/L mononuclear cells (normal < 6 × 10^6^/L), protein 0.27 g/L (0.15–0.42 g/L), neopterin 56.96 nmol/L (6–30 nmol/L) and negative gram stain, culture and PCR for bacterial and viral pathogens (Table S1). CSF opening pressure was >40 cm CSF (normal ≤ 25 cm CSF). Magnetic resonance imaging (MRI) brain and spine were normal (Fig. 1a1). Due to worsening headache and ongoing fever, CSF was repeated 1 week later and showed 610 × 10^6^/L polymorphs, 241 × 10^6^/L mononuclear cells, protein 1.18 g/L, neopterin 287.43 nmol/L and no growth on bacterial culture.

Tuberculosis meningitis was suspected as she migrated from India 4 months prior. She was treated with antituberculosis medications and prednisolone 2 mg/kg daily with a weaning schedule. Fevers settled and she was discharged home 6 weeks after symptom onset.

She re‐presented 10 days later with severe headache, Glasgow coma scale (GCS) score of 11, irritability, restricted right eye abduction, dysphasia and dysarthria, on prednisolone 10 mg daily and antituberculosis medications. Repeat CSF showed 1 × 10^6^/L polymorphs, 9 × 10^6^/L mononuclear cells and an elevated opening pressure 33.5 cm CSF. Investigations for tuberculosis remained negative and antitubercular drugs were stopped. Repeat MRI brain showed meningeal enhancement and T2/FLAIR hyperintensities with patchy enhancement of the basal ganglia, subcortical white matter and right cerebellar hemisphere (Fig. 1a2), and with the clinical progression, was consistent with ADEM. She was mycoplasma IgM positive and treated with azithromycin.

She received intravenous methylprednisolone (30 mg/kg daily for 5 days) with oral prednisolone tapering from 1 mg/kg over 2 months with resolution of symptoms. Within 24 hours of starting treatment, GCS improved to 15.

Serum MOG antibody was positive. After 2 months, she was asymptomatic with almost complete resolution of previous MRI changes. She remains well 18 months after discharge.

### Patient B

Patient B was a healthy 4‐year‐old female who presented to another institution with a 20‐min seizure with fever preceded by 5 days of abdominal pain and vomiting. She was treated with ceftriaxone for 2 days and discharged.

After 10 days, she had further seizures controlled with levetiracetam. After an ultrasound showing features of appendicitis, she underwent a laparoscopic appendicectomy and lumbar puncture (LP). Although macroscopic appearances of the appendix were normal, microscopic analysis was consistent with appendicitis. CSF showed 1 × 10^6^/L polymorphs, 6 × 10^6^/L mononuclear cells, protein 0.39 g/L and negative gram stain, culture and PCR for bacterial and viral pathogens (Table S1). CSF opening pressure was >32 cm CSF. MRI brain showed subtle bright FLAIR signal in the right frontoparietal cortex/subcortical white matter with associated meningeal enhancement (Fig. 1b1).

She was treated for infective meningoencephalitis with antibiotics and acyclovir but deteriorated with high fevers, drowsiness and irritability. Repeat CSF showed 125 × 10^6^/L polymorphs, 108 × 10^6^ mononuclear cells, protein 0.8 g/L, neopterin 371.37 nmol/L and negative gram stain with no growth. The patient was started on oral prednisolone 2 mg/kg with resolution of fever for 48 h before becoming febrile again.

Repeat MRI brain (2.5 weeks into her illness) showed multifocal T2/FLAIR hyperintensities (Fig. 1b2) consistent with ADEM. A speckled ANA pattern was detected at a titre of 80. Anti‐dsDNA antibodies and ENA screen were negative. She was treated with intravenous methylprednisolone (30 mg/kg daily for 5 days) and intravenous immunoglobulin 2 g/kg over 2 days. She defervesced within hours of starting methylprednisolone. Her drowsiness and irritability were resolved by completion of her methylprednisolone course. She was continued on a tapering prednisolone from 2 mg/kg daily over 4 months and discharged home after a 2.5‐week admission.

Serum MOG antibody was positive. She remains asymptomatic and well 4 months after discharge.

### Patient C

Patient C was a well 12‐year‐old girl who initially was admitted with an afebrile seizure and discharged after a normal electroencephalogram and head computed tomography. She was readmitted 10 days later with headache and papilloedema. The rest of the neurological examination was normal, including visual fields and visual acuity. Lumbar puncture opening pressure was 60 cm CSF. CSF showed 34 × 10^6^/L polymorphs, 113 × 10^6^/L mononuclear cells, protein 0.7 g/L, negative infection screen (Table S1) and neopterin 256.4 nmol/L. MRI brain was normal (Fig. 1c1). She was diagnosed with aseptic meningitis, treated with acetazolamide and discharged after 10 days of antibiotics and resolution of her headache.

She was readmitted 1 week later (1 month after the initial seizure) with ataxia, lower limb paraesthesia, intention tremor and urinary retention. Repeat LP showed 18 × 10^6^ polymorphs, 27 × 10^6^ mononuclear cells, 0.71 g/L protein, no growth and neopterin 151.9 nmol/L. MRI spine showed extensive T2 signal throughout the spinal cord consistent with longitudinally extensive transverse myelitis. MRI brain showed patchy, multifocal high T2 signal in the subcortical white matter and thalami suggestive of ADEM (Fig. 1c2). Speckled ANA pattern was detected at a titre of 320. ANCA, ENA screen, rheumatoid factor, anti‐dsDNA antibodies and anti‐GM1 antibodies were negative.

She was treated with intravenous methylprednisolone (30 mg/kg daily for 5 days), followed by prednisolone tapering from 1 mg/kg daily over 6 weeks with clinical and radiological resolution by completion of steroids. She was serum MOG antibody positive. She remains symptom‐free 7 years after presentation.

## Discussion

We present three cases of anti‐MOG‐associated aseptic meningitis preceding the development of ADEM or encephalomyelitis between 2 weeks and 2 months later. Initial brain MRIs were normal (2/3) or marginally abnormal. The diagnosis remained unclear until they developed clinical and radiological evidence of ADEM. Patients A and B briefly responded to prednisolone (2 mg/kg) but all patients progressed to ADEM or encephalomyelitis. Anti‐MOG antibody testing results became available weeks to years after initial presentations.

These cases add to two reports of paediatric MOGAD presenting with aseptic meningitis with progression to ADEM (Table 1).^3^, ^4^ All five patients rapidly responded to high‐dose methylprednisolone followed by weaning prednisolone. Our three patients had complete resolution without relapse (follow‐up between 4 months and 7 years). A recent article reported a further two children presenting with MOG‐associated aseptic meningitis and leptomeningeal enhancement, but clinical details were not published.^5^

**Table 1:** Clinical features of paediatric patients presenting with features of anti‐MOG associated aseptic meningitis

Patient	Age (years)	Sex	Features of the initial phase			Features after neurological deterioration				CSF opening pressure (cm CSF)	Treatment	Outcome (time from presentation)
			Symptoms	CSF (cells × 10^6^/L)	MRI	Time after onset	Symptoms	CSF	MRI			
A	5	Female	Fever and headache	6 polymorphs, 10 mononuclear cells, normal protein, neopterin 56.96 nmol/L	Normal brain and spine	5.5 weeks	Severe headache drowsiness, confusion and irritability	610 polymorphs, 241 mononuclear cells, protein 1.18 g/L, neopterin 287.43 nmol/L	Multifocal enhancing and non‐enhancing lesions in the deep grey matter, white matter and right cerebellar hemisphere	>40	Intravenous methylprednisolone (30 mg/kg daily) for 5 days and 2‐month oral prednisolone taper	No clinical sequelae (18 months)
B	4	Female	Abdominal pain, vomiting and seizure	1 polymorph, 6 mononuclear cells, normal protein	High FLAIR signal and meningeal enhancement of right frontoparietal cortex and subcortical white matter	2.5 weeks	High fevers, drowsiness and irritability	125 polymorphs, 108 mononuclear cells, protein 0.8 g/L, neopterin 371.37 nmol/L	Multifocal T2 and FLAIR hyperintensities in the white matter and deep grey matter	>32	Intravenous methylprednisolone (30 mg/kg daily) for 5 days, intravenous immunoglobulin 1 g/kg daily for 2 days and 4‐month oral prednisolone taper	No clinical sequelae (4 months)
C	12	Female	Seizure, then headache and papilloedema	34 polymorphs, 113 mononuclear cells, protein 0.7 g/L, neopterin 246.4 nmol/L	Normal brain	4 weeks	Ataxia, lower limb paraesthesia, intention tremor and urinary retention	18 polymorphs, 27 mononuclear cells, protein 0.71 g/L, neopterin 151.9 nmol/L	Multifocal T2 hyperintensities in the subcortical white matter, thalami and spinal cord	60	Intravenous methylprednisolone (30 mg/kg daily) for 5 days and 6‐week prednisolone taper	No clinical sequelae (7 years)
Leinert *et al*.^3^	6	Male	Fever, headache and neck stiffness	45 mononuclear cells, 11 polymorphs Protein 67.9 g/L	Not performed	3 days	Paraparesis and lower limb sensory changes, reduced right leg reflexes, positive Babinski sign bilaterally, urinary retention	Not performed	Multifocal enhancing and non‐enhancing lesions in the subcortical white matter, left cerebellar peduncle, and spinal cord	Not available	Intravenous methylprednisolone (30 mg/kg daily) for 5 days	Thigh paresis with new contrast‐enhancing MRI lesions (5 weeks)
Vibha *et al*.^4^	13	Female	Fever, headache, vomiting and seizure	Not performed	Not performed	2 months	Vision loss of the right, then left eye over 7 days	150 cells (70% neutrophils, 30% lymphocytes), protein 52 mg/dL	Multifocal T2/FLAIR hyperintensities with bilateral optic nerve and diffuse leptomeningeal enhancement	14	Intravenous methylprednisolone for 5 days followed by oral steroids	Normal left eye visual acuity, mild vision impaired of right eye 6/9 (1 month)

Although ADEM is the commonest presentation of paediatric MOGAD, other syndromes include optic neuritis, transverse myelitis, aquaporin‐negative neuromyelitis optica spectrum disease and non‐ADEM encephalitis.^2^, ^6^ Seizures preceding radiological identification of demyelination have been reported, as in our cases.^7^ MOGAD is typically steroid responsive but has an increased risk of relapse with rapid steroid weaning and can be associated with permanent neurological sequelae if inadequately treated.^8^ However, there is no evidence available to direct the duration of the steroid wean and represents an area for future research. Thus, early recognition and adequate steroid therapy are critical. Earlier, high‐dose steroid treatment in our cases may have prevented progression to ADEM, given their rapid improvements after treatment.

Aseptic meningitis presents a diagnostic dilemma for clinicians due to the variable aetiologies. A cause is not found in up to 80% of cases and investigations are typically limited to infectious testing and, in some cases, neuroimaging.^1^ The main focus of management is excluding culture‐negative bacterial meningitis.^1^ Our case series identifies anti‐MOG‐associated neuroinflammation as a rare, but treatable cause of aseptic meningitis.

Aseptic meningitis with raised ICP is an important early clinical biomarker of MOG antibody‐associated neuroinflammation. Given steroid responsiveness, empiric treatment with high‐dose corticosteroids and testing for anti‐MOG antibodies should be considered in children with persisting aseptic meningitis.

## Ethics Statement

Informed consent was obtained from all patients and their families for inclusion in this report.

## Supporting information


**Table S1** Microbial testing which was all negative on cerebrospinal fluid for patients A, B and C
