# Supplementary material for: Steroid‐responsive aseptic meningitis with raised intracranial pressure syndrome associated with myelin oligodendrocyte glycoprotein autoantibodies
Source: J Paediatr Child Health. 2022 Aug 24;58(12):2322–6. doi: 10.1111/jpc.16189 (PMC10087128; doi:10.1111/jpc.16189)
Supplement: Supplementary file 1 — Table S1 Microbial testing which was all negative on cerebrospinal fluid for patients A, B and C [file JPC-58-2322-s001.docx]

| **Microbial Investigations on CSF** | **Patient A** | **Patient B** | **Patient C** |
| --- | --- | --- | --- |
| Gram stain | 🗸 | 🗸 | 🗸 |
| Bacterial culture | 🗸 | 🗸 | 🗸 |
| Acid fast bacilli and tuberculosis culture | 🗸 | 🗸 | 🗸 |
| Fungal culture | 🗸 | 🗸 |  |
| Escherichia coli PCR | 🗸 | 🗸 |  |
| Haemophilus influenzae PCR | 🗸 | 🗸 |  |
| Listeria monocytogenes PCR | 🗸 | 🗸 |  |
| Neisseria meningitidis PCR | 🗸 | 🗸 | 🗸 |
| Streptococcus agalactiae PCR | 🗸 | 🗸 |  |
| Streptococcus pneumoniae PCR | 🗸 | 🗸 | 🗸 |
| Cytomegalovirus PCR | 🗸 | 🗸 |  |
| Enterovirus PCR | 🗸 | 🗸 | 🗸 |
| Herpes simplex virus | 🗸 | 🗸 | 🗸 |
| Human herpesvirus 6 PCR | 🗸 | 🗸 |  |
| Varicella zoster virus PCR | 🗸 | 🗸 |  |
| Human parechovirus PCR | 🗸 | 🗸 |  |
| Cryptococcus neoformans/gattii PCR | 🗸 | 🗸 | 🗸 |
| Mycobacterium tuberculosis |  |  | 🗸 |

**Supplementary Table 1: Microbial testing** which was all negative on cerebrospinal fluid for patients A, B and C
